# Hospitalized Pets as a Source of Carbapenem-Resistance

**DOI:** 10.3389/fmicb.2018.02872

**Published:** 2018-12-06

**Authors:** Fabio Gentilini, Maria Elena Turba, Frederique Pasquali, Domenico Mion, Noemi Romagnoli, Elisa Zambon, Daniele Terni, Gisele Peirano, Johann Dawid Daniel Pitout, Antonio Parisi, Vittorio Sambri, Renato Giulio Zanoni

**Affiliations:** ^1^Department of Veterinary Medical Sciences, University of Bologna, Bologna, Italy; ^2^Genefast Srl, Forlì, Italy; ^3^Department of Agricultural and Food Sciences, University of Bologna, Bologna, Italy; ^4^Ospedale: Veterinario I Portoni Rossi s.r.l., Bologna, Italy; ^5^Unit of Microbiology, University of Calgary and Calgary Laboratory Services, Calgary, AB, Canada; ^6^Istituto Zooprofilattico Sperimentale della Puglia e della Basilicata, Foggia, Italy; ^7^The Great Romagna Hub Laboratory, Pievesestina, Italy; ^8^Department of Experimental, Diagnostic and Specialty Medicine - DIMES, University of Bologna, Bologna, Italy

**Keywords:** one health, carbapenem resistance, *Acinetobacter radioresistens*, dogs and cats, hospitalization, veterinary tertiary care facilities, *bla*_NDM-1_*bla*_OXA-23_

## Abstract

The massive and irrational use of antibiotics in livestock productions has fostered the occurrence and spread of resistance to “old class antimicrobials.” To cope with that phenomenon, some regulations have been already enforced in the member states of the European Union. However, a role of livestock animals in the relatively recent alerts on the rapid worldwide increase of resistance to last-choice antimicrobials as carbapenems is very unlikely. Conversely, these antimicrobials are increasingly administered in veterinary hospitals whose role in spreading bacteria or mobile genetic elements has not adequately been addressed so far. A cross-sectional study was carried out on 105 hospitalized and 100 non-hospitalized pets with the aim of measuring the prevalence of carbapenem-resistant Gram-negative bacteria (GNB) colonizing dogs and cats, either hospitalized or not hospitalized and estimating the relative odds. Stool samples were inoculated on MacConkey agar plates containing 1 mg/L imipenem which were then incubated aerobically at 37°C ± 1 for 48 h. Isolated bacteria were identified first by Matrix-assisted laser desorption/ionization time-of-flight mass spectrometry and were confirmed by 16S rRNA sequencing. The genetic basis of resistance was investigated using PCR methods, gene or whole genome sequencing (WGS). The prevalence of pets harboring carbapenem-resistant bacteria was 11.4 and 1.0% in hospitalized and not-hospitalized animals, respectively, with an odds ratio of 12.8 (*p* < 0.01). One pet carried two diverse isolates. Overall, 14 gram-negative non-fermenting bacteria, specifically, one *Acinetobacter radioresistens*, five *Acinetobacter baumannii*, six *Pseudomonas aeruginosa* and two *Stenotrophomonas maltophilia* were isolated. The *Acinetobacter* species carried acquired carbapenemases genes encoded by *bla*_NDM-1_ and *bla*_OXA-23_. In contrast, *Pseudomonas* phenotypic resistance was associated with the presence of mutations in the *oprD* gene. Notably, inherent carbapenem-resistant isolates of *S. maltophilia* were also resistant to the first-line recommended chemotherapeutic trimethoprim/sulfamethoxazole. This study estimates the risk of colonization by carbapenem-resistant non-fermenting GNB in pets hospitalized in veterinary tertiary care centers and highlights their potential role in spreading resistance genes among the animal and human community. Public health authorities should consider extending surveillance systems and putting the release of critical antibiotics under more strict control in order to manage the infection/colonization of pets in veterinary settings.

## Introduction

Among the large quantities of antimicrobials given to animals each year, only a fractional amount is reserved to pets (Guardabassi et al., 2004; European Medicines Agency, 2015). The latter estimate is expected to be undervalued since injectable molecules and human specialties are not adequately taken into account (European Medicines Agency, 2015). Even more interesting, it is the qualitative point of view; indeed, whereas in husbandry almost exclusively “old class antibiotics” are used; in veterinary tertiary care facilities (VTCF) for pets, also antimicrobials critically important for human medicine as carbapenems are administered in certain critical cases. In humans, *Enterobacteriaceae* and non-fermenting gram-negative bacteria (GNB) *Pseudomonas aeruginosa, Acinetobacter* spp. and *Stenotrophomonas maltophilia* account for the majority of the pandemic carbapenem resistance threat. The occurrence of carbapenem-resistant bacteria in veterinary medicine has already been brought to the attention of the scientific community due to the severe impact that this phenomenon may cause (Endimiani et al., 2011; Shaheen et al., 2013; Stolle et al., 2013). Resistant organisms are transmitted among pets, owners and veterinary staff and, in this way, spread to the community. (Boerlin et al., 2001; Guardabassi et al., 2004; Leite-Martins et al., 2014; Yao et al., 2016).

To date, the assessment of public health risk posed by VTCFs has not adequately been addressed and only scarce but relevant information is available (Boerlin et al., 2001; Pulss et al., 2018). Certainly, veterinary hospitals share some risk factors with human hospitals, such as the use of antibiotics, complex treatments, prolonged hospitalization of critically ill or immunocompromised patients, and the presence of intensive care units. These factors favor nosocomial infections, and the exchange of antimicrobial-resistant bacteria and genetic determinants of resistance (Poirel et al., 2008). In human hospitals, a particular concern has recently arisen, based on the evidence that the spread of resistant bacteria is not restricted to clonal outbreaks but also due to asymptomatic carriage (Cerqueira et al., 2017).

With the aim to refine the focus of antibiotic resistance in pets hospitalized in VTCFs, a cross-sectional study was thus carried out to investigate the prevalence of carbapenem-resistant GNB in pets admitted to VTCFs. To this aim, dogs and cats admitted to two different VTCFs in the Bologna area (Northern Italy) were included. In addition, privately owned pets living in the same area and not hospitalized were also investigated and compared in order to assess the relative odds of being colonized after hospitalization.

## Materials and Methods

### Experimental Design and Animals

This cross-sectional study was carried out within the Bologna area from November 2014 to January 2015. Feces from 105 hospitalized pets (61 dogs and 44 cats) and 100 non-hospitalized pets (73 dogs and 27 cats) were collected. Hospitalized pets from two different facilities, both of which were characterized by the presence of intensive care units and referral practices, were included consecutively after at least 48 h of hospitalization. The non-hospitalized pets were from 100 distinct owners, had never been hospitalized and had not been given antibiotics for at least 2 months; they will hereafter be defined as “general population.” All the private owners of the animals considered in this study signed an informed consent form to authorize the rectal swab sampling.

### Isolation and Identification of Enteric Bacteria

Rectal swabs were used for collecting the fecal material from the pets and were immediately placed in the transport medium Cary-Blair Medium (Oxoid). Within 24 h, the swabs were soaked in 1 mL of sterile saline, vortexed, and 0.1 mL was inoculated on plates of MacConkey Agar (Oxoid) containing 1 mg/L imipenem (Sigma-Aldrich). The plates were incubated aerobically at 37°C ± 1 for 48 h.

All the different morphotypes of colonies grown in the same plate were subcultivated to obtain pure culture. Isolates were identified firstly by Matrix-assisted laser desorption/ionization time-of-flight mass spectrometry (bioMérieux) and successively confirmed by partial sequencing of the *16S rRNA* gene (Brosius et al., 1978).

### Antimicrobial Susceptibility

For all isolates, the minimum inhibitory concentrations (MICs) of imipenem and meropenem was determined using *E*-test strips (bioMérieux) following manufacturer instructions. *P. aeruginosa, S. maltophilia*, and *Acinetobacter* spp. were considered resistant to imipenem and meropenem at values ≥ 8 mg/L as suggested by the EUCAST (2016). The MIC of colistin were determined by broth microdilution using a breakpoint > 2 mg/L (EUCAST, 22 march 2016). Disk diffusion was used for testing all other antibiotics (Supplementary Material) and interpreted according to Clinical and Laboratory Standards Institute standard (CLSI 2014 M100-S24).

### Molecular Characterization of Antimicrobial Resistance

The isolates were screened for the presence of the following genes coding for carbapenem-hydrolyzing enzymes: *bla*_V IM_, *bla*_NDM-1_, *bla*_IMP_, *bla*_KPC_, and *bla*_OXA-48-like_ using a multiplex polymerase chain reaction (PCR) assay as previously described (Doyle et al., 2012). In addition, a real-time PCR was used for detecting *bla*_OXA_*_-23_* (Supplementary Material) followed by direct Sanger sequencing of the amplicons as a confirmatory assay. The presence of AmpC was carried out using a previously described multiplex PCR (Pérez-Pérez and Hanson, 2002) while extended-spectrum β-lactamases (ESBL) genes were detected using a multiplex PCR with subsequent melt curve analysis suitable to identify *bla*_SHV_, *bla*_TEM_, and *bla*_CTX-M_ gene targets (Supplementary Material).

Two PCR reactions were used to confirm the presence of chromosomally encoded metallo β-lactamases (MBL) in *S. maltophilia* (Supplementary Material); in particular, two PCR assays were carried out targeting an internal sequence of the *L1* gene and a sequence located within the region bridging the transcription factor encoding gene *ampR* and the inducible β-lactamase *L2* gene (Gould et al., 2006; Okazaki and Avison, 2008). Furthermore, trimethoprim/sulfamethoxazole (SXT) resistance was investigated using primers pairs for *sul1, sul2*, and *dfrA* genes as well as class 1, 2, and 3 integrons (Hu et al., 2011; Supplementary Material).

To investigate whether *bla*_NDM-1_ was located in transposon Tn*125*, a PCR was carried out at 5′ terminus with primers fwd_IS*Aba125* and NDM-reverse and at the 3′ terminus of the transposon with the fwd primer annealing on F_*Δpac* _and rev_IS*Aba125* (Supplementary Material). Finally, the *oprD* gene of *P. aeruginosa* was amplified using outer primers, amplifying the almost complete sequence of the gene followed by sequencing of the amplicon by internal primers (Supplementary Material).

### Whole Genome Sequencing

Since, to the best of our knowledge, *Acinetobacter radioresistens* carrying chromosomally encoded *bla*_NDM-1_ has not previously been reported, the whole genome of the isolate was sequenced to better characterize the resistance pattern. Also the five isolates of *A. baumannii* were fully sequenced to characterize the *bla*_OXA_*_-_*_23_ genetic environment, the complete resistome profile and to assess the Multi Locus Sequence Type as well as the clonal relationship. For Whole Genome Sequencing (WGS), genomic DNA was extracted from overnight broth culture in Brain Heart Infusion (Oxoid, Basingstoke, United Kingdom) using the MagAttract HMW DNA kit (Qiagen, Milan, Italy). The DNA samples were quantified with a fluorescent nucleic acid dye (Picogreen; Invitrogen, Paisley, United Kingdom), and libraries were prepared using the Nextera XT DNA Sample Preparation Kit (Illumina, Inc., San Diego, CA, United States). Sequencing was carried out on an Illumina MiSeq platform with 2 × 250 bp paired-end runs. The sequence quality of the reads was evaluated using FastQC (Andrews, 2016). The reads were assembled with SPAdes 3.7.1. The ResFinder web server^1^ using a threshold of 100% identity for the genes encoding β-lactamases and 98% identity for all other genes and CARD^2^ (Jia et al., 2017) using the Resistance Gene Identifier v 4.0.2 were used to search for antimicrobial resistance genes in the *Acinetobacter* spp. draft genomes.

### MLST

The Sequence Type of *A. baumannii* isolates was inferred from assembled contigs using the Multi Locus Sequence Typing application 1.8 (Assembled Genomes/Contigs) (Larsen et al., 2012).

In addition, to further analyze the genetic relatedness of the apparently clonal *A. baumannii* isolates, an alternative approach to the MLST, the wgMLST was carried out by genome-wide gene-by-gene comparisons of a dataset of 5633 genes retrieved from the whole genome assemblies using the Bionumerics 7.6 package software (Applied Maths) using either an assembly free and an assembly based algorithm in Bionumerics engine calculation. The isolate 92A IMI was assumed as index isolate for comparison. The results were graphed as dendrogram with percentage of homology (Supplementary Figure S1).

### PFGE Typing

For clonality assessment*, A. baumannii* isolates were typed using a rapid PFGE protocol with *Apa*I (40 U/plug) (Promega) as a restriction enzyme according to Durmaz (Durmaz et al., 2009).

### Nucleotide Accession Number

The raw sequence data of *Acinetobacter radioresistens* and of five *Acinetobacter baumannii* isolates were deposited in the GenBank database under BioProject accession PRJNA344732 and PRJNA437120, respectively.

## Results

Thirteen out of 205 pets carried carbapenem-resistant bacteria with a prevalence estimate of 6.3% [95% confidence interval (CI): 3.4–10.6%]. Of these, 12 (8 dogs and 4 cats) were hospitalized pets [prevalence estimate 11.4% (95% CI: 6.0–19.1%)] and 1 dog was from the general population [prevalence estimate 1.0% (95% CI: 0.0–5.4%)]. The odds ratio of the hospitalized pets was 12.8 (95% CI: 1.6–100.2; *p* < 0.01). In one hospitalized cat, two different carbapenem-resistant bacteria were isolated; therefore, 14 isolates were found in total. They included one *Acinetobacter radioresistens*, five *A. baumannii*, six *P. aeruginosa*, and two *S. maltophilia* (Supplementary Table S1). The MICs of imipenem for the *Acinetobacter* species ranged from 16 mg/L to more than 32 mg/L, for the *P. aeruginosa* they were 16 mg/L and for the *S. maltophilia* they were > 32 mg/L (Table 1). All bacteria showed multiple resistance, however, in all cases, the bacteria were susceptible to colistin (MIC < 2 mg/L). Carbapenemase resistance genes were detected in all isolates except *P. aeruginosa.*

**Table 1:** Characteristics of the isolates resistant to carbapenems obtained from feces of dogs and cats.

Isolate	Organism	MIC of imipenem (mg/L)	MIC of meropenem (mg/L)	Resistance determinant		Dog/Cat (source)	Hospitalized
						
				Carpapem-resistance	Other β-lactamases		
1A IMI	*Acinetobacter radioresistens*	>32	>32	*bla*NDM-1	*bla*OXA-23	Dog	Yes
87A IMI	*Acinetobacter baumannii* (ST2)	>32	>32	*bla*OXA-23	*bla*OXA-66*,bla*TEM, AmpC	Cat	Yes
92A IMI	*Acinetobacter baumannii* (ST2)	16	>32	*bla*OXA-23	*bla*OXA-66,*bla*TEM, AmpC	Dog	Yes
108A IMI	*Acinetobacter baumannii* (ST2)	16	>32	*bla*OXA-23	*bla*OXA-66,*bla*TEM, AmpC	Cat	Yes
115A IMI	*Acinetobacter baumannii* (ST2)	>32	>32	*bla*OXA-23	*bla*OXA-66,*bla*TEM, AmpC	Dog	Yes
213A IMI	*Acinetobacter baumannii* (ST2)	16	>32	*bla*OXA-23	*bla*OXA-66,*bla*TEM, AmpC	Cat^∗^	Yes
3 A IMI	*Pseudomonas aeruginosa*	16	8	oprD	*bla*SHV AmpC	Dog	Yes
110A IMI	*Pseudomonas aeruginosa*	16	4	oprD	*bla*SHV CTX-M	Dog	Yes
111A IMI	*Pseudomonas aeruginosa*	16	8	ND	*bla*SHV	Dog	Yes
117A IMI	*Pseudomonas aeruginosa*	16	2	oprD	*bla*TEM	Dog	Yes
121A IMI	*Pseudomonas aeruginosa*	16	4	oprD	AmpC	Dog	Yes
131A IMI	*Pseudomonas aeruginosa*	16	8	ND	*bla*SHV AmpC	Cat	Yes
207A IMI	*Stenotrophomonas maltophilia*	>32	>32	L1		Dog	No
213B IMI	*Stenotrophomonas maltophilia*	>32	>32	L1		Cat^∗^	Yes


*MIC, Minimum Inhibitory Concentration; ND, not determined; ST, Sequence type (Pasteur scheme). ^∗^Indicates the same animal.*

**FIGURE 1:**
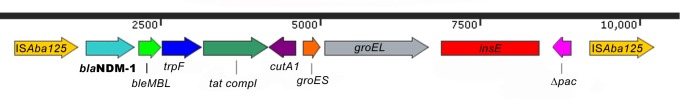
*Acinetobacter radioresistens bla*_NDM-1_ genetic environment. The schematic presentation of the *A. radioresistens bla*_NDM-1_ transposon Tn*125* encompassing *bla*_NDM-1_, *bleMBL, trpF, tat, cutA1, groES, groEL, insE*, and *Δpac* genes bracketed between a pair of IS*Aba125* is drawn above. Red rectangles indicate the position of the amplicons.

*A. radioresistens* carried both *bla*_NDM-1_ and *bla*_OXA-23._ The *bla*_NDM-1_ gene was chromosomally located and organized in a composite transposon encompassing *bla*_NDM-1_, *bleMBL, trpF, tat, cutA1, groES, groEL, insE*, and *Δpac* genes bracketed between a pair of IS*Aba125*. The presence of a mobile element with IS elements was further confirmed by a specific PCR assay designed to investigate the genetic environment of *bla*_NDM-1_ gene (Figure 1).

The *bla*_OXA-23_ lacked IS*Aba1*, IS*Aba4* (Turton et al., 2006; Corvec et al., 2007; Poirel et al., 2008; Karthikeyan et al., 2010; Smet et al., 2012) or IS*Acsp2* (Poirel et al., 2012) upstream insertion sequences according to ISfinder^3^. These IS sequences are known to provide the effective promoter to the hydrolyzing enzyme encoding gene *bla*_OXA-23_ in *A. baumannii* for an elevated level of expression (Turton et al., 2006). Other genetic determinants of resistance included aminoglycoside resistance encoding genes *strA* [aph(3″)-ib] and *strB* [aph(6)-1d], sulphonamide resistance gene *sul2*, bleomycin resistant protein BRP and tetracycline resistance gene *tet*.

The *A. radioresistens* whole genome was sequenced, and the reads showed a coverage of 293X and a guanine-cytosine (GC) content of 42%. The assembled genome encountered 81 contigs with N50 of 113445 and mean contig size of 38028.383 bp. The genome length was 3.080 Mbp.

**FIGURE 2:**
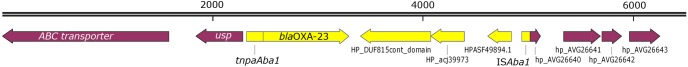
*Acinetobacter baumannii bla*_OXA-23_ genetic environment. The schematic presentation of the *A baumannii bla*_OXA-23_ transposon Tn*2006* encompassing *bla*_OXA-23_, and few ORFs bracketed between IS*Aba1* colored in yellow. Flanking ORFs are colored in purple.

All the *A. baumannii* strains carried both intrinsic *bla*_OXA-66_ and acquired *bla*_OXA-23_ Class D Oxacillinases. However, while *bla*_OXA-23_ was embedded in a transposon bracketed within two IS*Aba1* insertion sequence which provide a strong promoter leading to a sustained expression of oxacillinase hydrolyzing enzyme, the *bla*_OXA-66_ lacks an upstream IS sequence (Figure 2).

Additionally, besides oxacillinases, other β-lactamases were present in all isolates of *A. baumannii.* Acinetobacter derived cephalosporinase *bla*_ADC-73_ (AmpC allele 20) with an upstream IS*Aba1* in the promoter region and a likely sustained expression was present. Furthermore, *A. baumannii* isolates carried *bla*_TEM-1D_ embedded in a single copy IS*26*- *bla*_TEM-1D_-Tn3-IS*26*, likely responsible for the sulbactam resistance phenotype (Krizova et al., 2013; Yang et al., 2018).

Overall, the antimicrobial resistance profile of the five *A. baumannii* isolates showed an extensively drug-resistant (XDR) phenotype, i.e., non-susceptibility to at least one agent in all but two or fewer antimicrobial categories (Supplementary Table S2; Magiorakos et al., 2012). The comprehensive resistance determinants are enlisted in Supplementary Table S3.

The two *S. maltophilia* isolates carried inducible chromosomal encoded *L1* metallo-β-lactamase with carbapenem hydrolyzing activity and its regulatory element *ampR* (Gould et al., 2006; Okazaki and Avison, 2008). Notably, both *S. maltophilia* isolates were resistant to SXT due to the presence of both *sul1* and *sul2* genes associated with class 1 integron mobile element.

Finally, carbapenem-resistant *P. aeruginosa* were negative to carbapenemase genes but carried either *bla*_SHV_, and/or *bla*_TEM_, and/or *bla*_CTX-M_ β-lactamase associated with an *oprD* gene with truncating mutations in 4 out of 6 of these isolates (Tables 1, 2).

**Table 2:** *oprD* mutations in *Pseudomonas aeruginosa* isolates resistant to carbapenems.

Isolate	*oprD* mutation	Resulting change in the protein
3A IMI	c.604ins (IS256 family transposase ISPa1328)	Protein truncated
110A IMI	c.840_854delGGGCGCGTATACCCTinsCGGC	Frameshift and premature stop codon; Predicted protein of 313 aa
111A IMI	c.875 T > C	p.292P > L
117A IMI	c.970delA	Frameshift and premature stop codon; Predicted protein of 344 aa
121A IMI	c.1244G > A	Nonsense mutation and premature stop codon; Predicted protein of 414 aa
131A IMI	c.154G > A; c.397A > C;	p.52D > N p.132K > Q


*Strain used for comparison: P. aeruginosa ATCC 27853 (GenBank Accession Number KF649209.1). Missense mutations with respect to the reference strain are indicated only in those isolates without truncating mutations.*

All *A. baumannii* strains showed the same macrorestriction profile by PFGE (Supplementary Figure S2). All isolates were assigned to ST2 (Pasteur’s scheme) or ST451 (Oxford’s scheme). Using the complete panel of the coded alleles a UPGMA tree was calculated in Bionumerics 7.6 software (Supplementary Figure S1). The wgMLST showed that all isolates except one were closely related with homology > 99.8%. (<6 diverse allele). The only isolate 108A IMI seemed more dissimilar with an homology of 98.8%. The most closely related isolates were sampled on the same day (87A IMI and 92A IMI) or 2 months apart (115A IMI and 213A IMI) (Supplementary Figure S1).

## Discussion

This study findings focuses on *Acinetobacter* spp. as the main emerging threat to public health associated with the hospitalization in VTCFs. Notably, we identified for the first time to the best of our knowledge, a carbapenem resistant *A. radioresistens* isolate carrying a *bla*_NDM-1_ gene within a *Tn*125 mobile element. The same composite *Tn*125 has already been reported in other *Acinetobacter* species (Pfeifer et al., 2011; Bonnin et al., 2012; Dortet et al., 2012; Fu et al., 2012; Chatterjee et al., 2016) but not yet in *A. radioresistens*. The presence of a Class B MBL in a mobile element is a concern since it could be readily transferred to other bacteria residing in the intestine, such as *Enterobacteriaceae*. Its potential to serve as a reservoir for worldwide dissemination of *bla*_NDM_ should be given outstanding attention and should not be undervalued in view of the fact that was isolated in a veterinary and not human tertiary care settings.

Conversely, *A. radioresistens* was found to carry* bla*_OXA-23_ without IS*Ab1* sequences and, as a consequence, the hydrolyzing oxacillinase was not expressed at high level. Overall, *A. radioresistens* was multi-drug resistant being not susceptible to quinolones, tetracycline and trimethoprim/sulfamethoxazole though it remained susceptible to amikacin and gentamicin, which are not affected by Aminoglycoside O-phosphotransferases *strA* and *strB*, as well as to ampicillin/sulbactam and colistin.

Noteworthy, the resistome of the five *A. baumannii* isolates showed an XDR phenotype (Supplementary Table S2) including non-susceptibility to quinolones, gentamicin, amikacin and sulbactam with only colistin as putative effective treatment. It is worth mentioning that also *16S rRNA methylase* gene *armA* recently reported in nosocomial outbreaks in Italy and responsible for resistance to aminoglycosides including gentamicin and amikacin, was found (Brigante et al., 2012). For these features, the *A. baumannii* isolates represent an actual hazard retaining high epidemic potential.

*A. baumannii* is reported as a frequent cause of nosocomial infections with increased mortality worldwide (Higgins et al., 2010). Very recently, single cases and small case series of clinical isolates of carbapenem resistant *A. baumannii* of different sequence types and carrying *bla*_OXA-23_ were reported (Pomba et al., 2014; Ewers et al., 2016, 2017; Lupo et al., 2017). Those authors focused the attention on companion animals as potential public health hazard. Also a remarkably high rate of asymptomatic pet carriers of carbapenem-resistant *A. baumannii* ST25 (2.7%) has been reported in France and pointed out pets as possible reservoir of community acquired infections (Hérivaux et al., 2016).

All the *A. baumannii* isolates in VTCFs in our study belong to ST2. In Italy, besides, the endemic so called “Italian strain” ST78, the ST2 strains belonging to International Clonal lineage II has progressively become dominant as a cause of epidemic outbreaks in hospitals and is even replacing the ST78 strains. Both strains share great ability to adhere to cells, to invade and survive within pneumocytes as well as to form biofilm on abiotic surface (Ambrosi et al., 2017) hence, replacing of the ST78 may be associated with the acquisition of ST2 strains of highly expressed *bla*_OXA-23_ oxacillinase (D’Arezzo et al., 2011; Brigante et al., 2012; Mezzatesta et al., 2012). Indeed, in last years, nosocomial outbreaks in Italy are almost all ascribed to ST2 *bla*_OXA-23_ producing strains (Carretto et al., 2011; Agodi et al., 2014; Principe et al., 2014; Perilli et al., 2015; Piana et al., 2015). *Bla*_OXA-23_ producing strains were not reported in a survey, which had investigated nosocomial *A. baumannii* isolated in 2007 in the Bologna Area, while they were reported in another survey carried out later in 2011 (Carretto et al., 2011; Principe et al., 2014).

W*ithin*-hospital selective pressure clearly plays a role in favoring the emergence of MDR but there is also *inter*-hospital interconnection modalities (i.e., patients transfer) which explains the simultaneous outbreaks of related resistant *A. baumannii* strains (Karkada et al., 2011). How the VTCFs/ICUs fit in this model is far from being understood but the evidence that the strains isolated in veterinary and human medical settings in the same geographical area worth to be examined as well as the role of the community.

When investigating the clonal relatedness, all *A. baumannii* isolates showed the same PFGE pattern. Also wgMLST confirmed the high relatedness of at least of four of five of the isolates whereas one isolate showed less similarity, however, the complexity in interpreting the genomic variability is challenging, since no threshold guidelines could be applied and a case-by-case interpretation is still used in this matter (Halachev et al., 2014; Higgins et al., 2017; Shaheen et al., 2018); Since this study did not deal with an epidemic outbreak, the *A. baumannii* isolates found during the survey may represent the genetic evolution over time of originally the same clone which has colonized the medical environment for its ability to grow on abiotic surface, has accumulated genetic variations making it to diverge from the original clone. Eventually, the knowledge of the frequency rate of mutation accumulation in *A. baumannii* would lead to infer the precise time of colonization.

Unlike *Acinetobacter* isolates, *P. aeruginosa*-associated carbapenem resistance is linked to the loss of oprD (Lee and Ko, 2012; Ocampo-Sosa et al., 2012; Kim et al., 2016) and it is associated with multidrug resistance. In most of our cases, the loss of oprD was caused by different mutations within the gene causing a premature stop codon as a consequence of a large insertion, a frameshift or a nonsense mutation. The loss of oprD was also associated with either ESBL (*bla*_SHV_, *bla*_TEM_ or *bla*_CTX-M_) or AmpC resistance genes. The presence of different mutations and β-lactamase genes indirectly demonstrates the lack of clonal relatedness at least between the *P. aeruginosa* isolates. The absence of clonal spread in apparent pseudo-outbreaks is very frequent for Gram-negative non-fermenting bacteria and it is correlated to their ability to colonize the medical device and surface (Valdezate et al., 2004) and produce multispecies biofilms (Tan et al., 2017).

Together with *Acinetobacter* spp. and *P. aeruginosa*, other carbapenemase-resistant non-fermenter GNB, namely *S. maltophilia*, were found. *S. maltophilia* is not an inherently primary pathogen, but it is rather environmental bacteria intrinsically resistant to β-lactams including carbapenems due to inducible β-lactamase (Gould et al., 2006; Okazaki and Avison, 2008). It should be noted that, in *S. maltophilia* human nosocomial infections, the antibiotic of choice for treatment of infections caused by these bacteria is SXT (Madi et al., 2016). Our isolates actually showed complete resistance to SXT. Both *sul1* associated with class 1 integron, and *sul2* genes were found in the isolates. Plasmid-mediated *sul1* and *sul2* are responsible for the worldwide emergence of resistance to sulfonamides reported beginning since 2007 (Toleman et al., 2007). In 2009, a comprehensive meta-analysis carried out on 3872 strains, found 4% of SXT resistant strains (Looney et al., 2009). As far as we know, the only report from southern Europe, namely from Greece, showed that the majority of *S. maltophilia* clinical isolates were sensitive to SXT (Samonis et al., 2012) while most of the concerns were from Middle east (Turkey, Iran) and Far East (Korea and China) (Liaw et al., 2010). The origin of this resistance at the genetic level was ascribed to *sul* genes. In particular, the presence of *sul2* is reported in isolates showing very high resistance to SXT (Toleman et al., 2007). However, *sul* genes alone do not confer trimethoprim resistance but certainly, they are frequently associated with *dfrA* genes in the gene cassettes of the integrons. In our isolates, *dfrA* genes were not found and the high resistance to SXT was not clarified. Furthermore, class 1 integrons may carry genes coding for tolerance to common disinfectants and facilitate the horizontal spread of resistance to other bacterial species. Overall, these features make these opportunistic pathogens isolated in this study an insidious threat.

On a more general note, the findings of the study show that hospitalization in VTCFs providing cures to pets represents a significant risk factor of colonization with carbapenem resistant GNB embodying the most significant current hazard to public health in VTCFs. These findings are even more worrying if compared with those found in human hospitals in very similar cross-sectional epidemiological survey during non-epidemic outbreaks. The carbapenemase producing GNB was 0.4% compared with the 6% of this study and the overall prevalence of carbapenem-resistant GNB was 4.8% compared with the 11.4% of this study (Pantel et al., 2015). The other side of the coin, thankfully, is that the more pathogenic *Enterobacteriaceae* carrying carbapenemase resistance genes reported to cause epidemic cases around the world and also recently found in Germany in many different veterinary hospitals (Pulss et al., 2018) were not found in these veterinary healthcare settings. It should be noted that KPC producers (mainly *Enterobacteriaceae*) may exhibit susceptibility to imipenem and resistance to ertapenem causing an underestimation of their prevalence using MacConkey supplemented with imipenem (1 mg/L) as carried out in this study. However, KPC producers were not shown either using ceftazidime (1 mg/L) for ESBL bacteria nor using multiplex PCR directly on the DNA purified from stools for molecular screening (data not shown).

Non-fermenter GNB may cause nosocomial infections, particularly in immunocompromised critically ill patients, raising the mortality rate (Siempos et al., 2010; Chiu et al., 2015). Furthermore, *Acinetobacter* spp. and *S. maltophilia*, but also *P. aeruginosa*, show great ability to adapt to any environment, including hospitals; the clonal relatedness between the isolates of *A. baumannii* carried by pets hospitalized in one VTCF over a short period of time demonstrated the colonization of the environment. Non-fermenter GNB are also readily carried on the skin of colonized animals thus enabling the easy transmission to veterinarians, personnel providing care and owners as well as other hospitalized pets. These bacteria represent a rising challenge for healthcare management because they are frequently multidrug resistant as a consequence of a multitude of mechanisms. In addition, non-fermenter GNB may be a source of genes which are carbapenemase resistant for *Enterobacteriaceae* (Toleman et al., 2012; Bonnin et al., 2014). It should be emphasized that non-fermenter GNB were not isolated in clinical samples, but they colonized the intestines of the pets; no outbreaks sustained by non-fermenter GNB were reported in either of the investigated VTCFs during the period of study and, hence, no awareness of the risk was evident and no specific measures of control were activated.

In VTCFs, the concerns of carbapenem resistance should be even more compelling with respect to human hospitals due to the extensive, systematic use of broad-spectrum antibiotics, the relative lack of antimicrobial susceptibility-based therapy, the objective difficulties in applying and maintaining hygienic practices and the very limited use of systematic monitoring programs. In fact, these findings and considerations give rise to concerns regarding the risk for veterinary personnel veterinary students or trainees and for the pet owners, highlighting the possible role that veterinary facilities play in spreading carbapenemase-resistant genes in the environment, including the human community.

In the VTCFs investigated in this study, wide spectrum β-lactam drugs are used as first line therapy in hospitalized dogs. The consequent high environmental pressure of such antibiotics represents a risk factor for the co-selection of carbapenem-resistant bacteria (Maseda et al., 2017). In fact, almost all the carbapenem-resistant isolates are either Sulbactam resistant or AmpC or ESBL producers (data not shown), i.e., the “restricted use” of a specific class of antibiotics is not *per se* sufficient to avoid the emergence of the relative resistance. Since it is evident that carbapenem-resistant gram negative non-fermenters represent an actual threat in VTCFs, it appears more reasonable to also extend the public health surveillance system to VTCFs, and to allow the traceable and targeted use of carbapenems or other critical antibiotics, such as colistin, to control infection or to eradicate the carriage of multidrug resistant bacteria in pets. Appropriate antibiotic stewardships as well as active and continuous surveillance of resistance should be implemented in VTCFs which ask to be accredited for the use of carbapenems or last-choice antimicrobials.

## Author Contributions

FG and RZ provided substantial contributions to the conception and design of the work and the acquisition, analysis, and interpretation of data and drafting the work. FP and DM performed the acquisition, analysis, and interpretation of data and drafting the work. VS and AP performed the acquisition, analysis, and interpretation of data and critical revising of the work for important intellectual content. MT, NR, EZ, DT, GP, and JP performed the acquisition and analysis of data and critical revising of the work for important intellectual content. All authors have approved the final version to be published. All authors are to be accountable for all aspects of the work in ensuring that questions related to the accuracy or integrity of any part of the work are appropriately investigated and resolved.

## Conflict of Interest Statement

The authors declare that the research was conducted in the absence of any commercial or financial relationships that could be construed as a potential conflict of interest.

## Footnotes

1 www.genomicepidemiology.org

2 https://card.mcmaster.ca

3 www-is.biotoul.fr
