# Supplementary material for: Hospitalized Pets as a Source of Carbapenem-Resistance
Source: Front Microbiol. 2018 Dec 6;9:2872. doi: 10.3389/fmicb.2018.02872 (PMC6291488; doi:10.3389/fmicb.2018.02872)
Supplement: Supplementary file 1 [file Data_Sheet_1.docx]

**^§^Primers designed in this study**

**PCR methods**

**ESBL qPCR and Melting Curve analysis**

*Primers*

FWD_SHVu 5’-GCAAAACGCCGGGTTATTC-3’

^§^R_SHVin 5’-GCCGGCGTATCCCGCAGATAA-3’

FWD_CTX-Mu 5’-CGCTTTGCGATGTGCAG-3’

REV_CTX-Mu 5’-ACCGCGATATCGTTGGT-3’

^§^F_Tem_in 5’-TGCTCACCCAGAAACGCTGGT-3’

^§^R_Tem_in 5’-TGCTGCAGGCATCGTGGTGT-3’

*Reaction mix*

qPCR Master Mix (Promega) 1X, , forward and reverse *bla*SHV primers 300 nM each, forward and reverse *bla*CTX-M primers 150 nM, forward and reverse *bla*TEM primers 250 nM, 1µL of template DNA, Water molecular grade reagent up to 25 µL

*Amplification protocol*

95°C for 2 min, followed by 40 cycles composed of denaturation at 95°C for 20 s, annealing at 60°C for 30s and extension at 72°C for 1.30 min followed by melt curve analysis.

*bla*SHV family: T_m_ ≈ 90°C

*bla*CTX-M family: T_m_ ≈ 89°C

*bla*TEM family: T_m_ ≈ 84°C

**
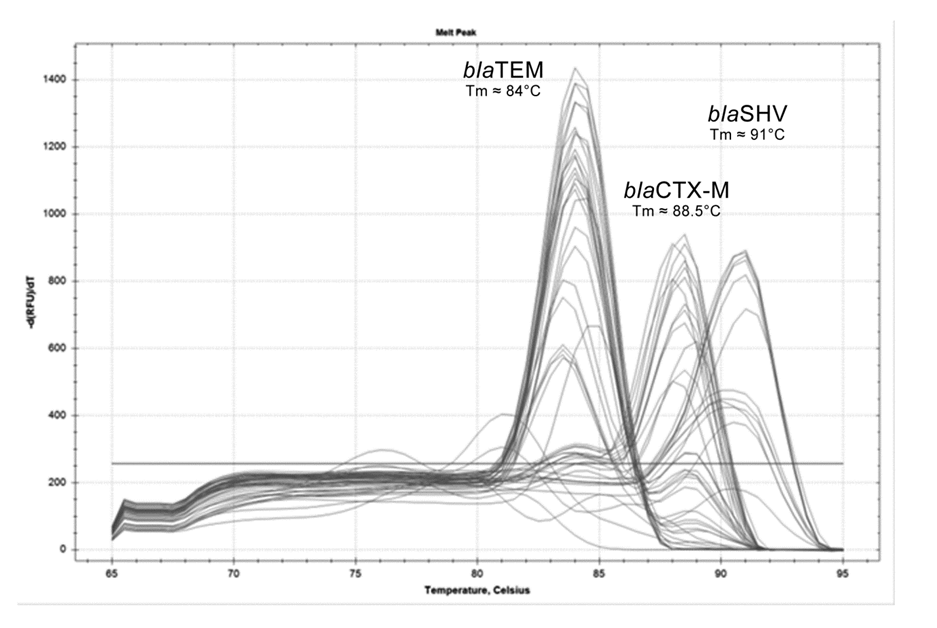
**

**L1**

*Primers (Primer Express software v2.0, Thermo Fisher Scientific)*

^§^F_sm_L1 5’-GGCGACGGCATCACCTAT-3’

^§^Rev__bis_L1_sm 5’-CTTGCCATCGCGGGTATCGG-3’

*Reaction mix*

Phusion (Thermo Scientific) 0.4u, HF buffer 1X, dNTPs 200 µM each, additional Mg 0.25mM, forward and reverse primers 400 nM each, 1µL of template DNA, Water molecular grade reagent up to 25 µL

*Amplification protocol*

98°C for 30 s, followed by 40 cycles composed of denaturation at 98°C for 10 s, annealing at 69°C for 10s and extension at 72°C for 10 s.

**AmpR-L2**

*Primers (Primer Express software v2.0, Thermo Fisher Scientific)*

^§^F_sm_AmpR_L2_good 5’-CGGGTGAAGTTCTGGTGGCG-3’

^§^R_sm_AmpR_L2_best 5’-CTGCAGGAATCGGCGACGG-3’

*Reaction mix*

Phusion (Thermo Scientific) 0.4u, HF buffer 1X, dNTPs 200 µM each, additional Mg 0.5mM, forward and reverse primers 400 nM each, 1µL of template DNA, Water molecular grade reagent up to 25 µL

*Amplification protocol*

98°C for 30 s, followed by 40 cycles composed of denaturation at 98°C for 10 s, annealing at 71.6 °C for 10 s and extension at 72°C for 10s.

**SXT resistance in *S. maltophilia***

*Class 1 integrase*

Temperature of annealing: 59.3°C

intI1-R 5’-CAGTGGACATAAGCCTGTTC-3’

intI1-F 5’-CCCGAGGCATAGACTGTA-3’

*Class 1 integron*

Temperature of annealing: 54.1°C

In1-R 5’-AAGCAGACTTGACCTGA-3’

In1-F 5’-GGCATCCAAGCAGCAAG-3’

*Class 2 integron*

Temperature of annealing: 65.7°C

hep74 5’-CGGGATCCCGGACGGCATGCAC-3’

hep51 5’-GATGCCATCGCAAGTACGAG-3’

*Class 3 integrase*

Temperature of annealing: 64.0°C

int3-R 5’-TGTTCTTGTATCGGCAGGTG-3’

int3-F 5’-AGTGGGTGGCGAATGAGTG-3’

*sul1*

Temperature of annealing: 59.3°C

sul1-R 5’-ATTCAGAATGCCGAACACCG-3’

sul1-F 5’-TAGCGAGGGCTTTACTAAGC-3’

*sul2*

Temperature of annealing: 66.6°C

sul2-R 5’-GAAGCGCAGCCGCAATTCAT-3’

sul2-F 5’-CCTGTTTCGTCCGACACAGA-3’

dfrA1

Temperature of annealing: 61.0°C

dfrA1-R 5’-TTGTGAAACTATCACTAATGGTAG-3’

dfrA1-F 5’-CTTGTTAACCCTTTTGCCAGA-3’

dfrA5

Temperature of annealing: 61.0°C

dfrA5-R 5’-TCCACACATACCCTGGTCCG-3’

dfrA5-F 5’-ATCGTCGATATATGGAGCGTA-3’

dfrA12

Temperature of annealing: 63.8°C

dfrA12-R 5’-ATGAACTCGGAATCAGTACGC-3’

dfrA12-F 5’-TTAGCCGTTTCGACGCGCAT-3’

dfrA13

Temperature of annealing: 58.6°C

dfrA13-R 5’-GAAACTATCACTAATGGCAGC-3’

dfrA13-F 5’-CTCATCTGCTGGCTATCTCA-3’

dfrA17

Temperature of annealing: 65.9°C

dfrA17-R 5’-TTGAAAATATTATTGATTTCTGCAGTG-3’

dfrA17-F 5’-GTTAGCCTTTTTTCCAAATCTGGTATG-3’

*Reaction mix*

Phusion (Thermo Scientific) 0.3u, HF buffer 1X, dNTPs 200 µM each, additional Mg 0.25mM, forward and reverse primers 500 nM each, 1µL of template DNA, Water molecular grade reagent up to 25 µL

*Amplification protocol*

98°C for 30 s, followed by 40 cycles composed of denaturation at 98°C for 10 s, annealing specific for each primer pair (listed above) °C for 15 s and extension at 72°C for 20 s.

**Transposon Tn*125***

5’-terminus

*Primers (Primer3Plus, http://www.bioinformatics.nl/cgi-bin/primer3plus/primer3plus.cgi)*

^§^Fwd_ISAba125 5’-GGCAGAATCAGTGCGCAAGGC-3’

^§^NDM-reverse 5’-GTGCTCAGCTTCGCGACC-3’

3’-terminus *(Primer3Plus, http://www.bioinformatics.nl/cgi-bin/primer3plus/prmer3plus.cgi)*

^§^F_∆pac 5’-TGACTGAACCAACTGTGAGTCCT-3’

^§^rev_ISAba125 5’-TTGAACTTCGGCTGGGCGCT-3’

*Reaction mix*

Hot Start GoTaq (Promega) 1 u, buffer 1X, dNTPs 200 µM each, Mg 2 mM, forward and reverse primers 400 nM each, 1µL of template DNA, Water molecular grade reagent up to 25 µL

*Amplification protocol*

95°C for 2 min, followed by 40 cycles composed of denaturation at 94°C for 30 s, annealing at 58°C for 30s and extension at 72°C for 1 min.

***bla*Oxa-23**

*Primers (Primer Express software v2.0, Thermo Fisher Scientific)*

^§^F_Oxa-23 5’-GGAGAACCAGAAAACGGAT-3’

^§^R_Oxa-23 5’-ATGGTCCTACCAACCAGA-3’

*Reaction mix*

GoTaq® qPCR Master Mix with CXR reference dye (Promega) 1 X, forward and reverse primers 400 nM each, 1µL of template DNA, Water molecular grade reagent brought to 20 µL

*Amplification protocol*

95°C for 2 min, followed by 40 cycles composed of denaturation at 95°C for 15 s, annealing and extension at 60°C for 1 min. followed by Melt curve analysis.

**OprD**

*Primers (Primer3Plus, http://www.bioinformatics.nl/cgi-bin/primer3plus/prmer3plus.cgi)*

^§^F_5_oprD 5’-ACTAGCCGTCACTGCGGCAC-3’

^§^R_3_oprD 5’-CGCGTTGCCGCCGAGAAGAA-3’

*Reaction mix*

Hot Start GoTaq (Promega) 1 u, buffer 1X, dNTPs 200 µM each, Mg 2 mM, forward and reverse primers 300 nM each, 1µL of template DNA, Water molecular grade reagent up to 25 µL

*Amplification protocol*

95°C for 2 min, followed by 40 cycles composed of denaturation at 94°C for 20 s, annealing at 58°C for 20s and extension at 72°C for 2 min.

^§^R_5_oprD 5’-CGGCGAAGACCGGAGCGG-3’

^§^F_3_oprD 5’-GGTCCATGGCGATCAGCCGTTT-3’

^§^F_OprD_seq_a 5’-CGAAGGCAAGCAGGGCACCA-3’

^§^F_OprD_seq_b 5’-TCAGCGCCTCCCTGTACGGT-3’

^§^F_OprD_seq_c 5’-AGGCCGGCGACATCAGCAAC-3’

^§^F_5'rev_OprD 5’-CCGCTCCGGTCTTCGCCG-3’
