# Supplementary material for: Hospitalized Pets as a Source of Carbapenem-Resistance
Source: Front Microbiol. 2018 Dec 6;9:2872. doi: 10.3389/fmicb.2018.02872 (PMC6291488; doi:10.3389/fmicb.2018.02872)
Supplement: Supplementary file 2 [file Data_Sheet_2.docx]

Captions

**Supplementary Figure 1**: Dendrogram representing the whole genome Multi Locus Sequence Typing of the five isolates of *A. baumannii*. The percentages of homology were indicated.

**Supplementary Figure 2**: PFGE macrorestriction profile of the five *A. baumannii* isolates.
