# Supplementary figures and images for: Hospitalized Pets as a Source of Carbapenem-Resistance
Source: Front Microbiol. 2018 Dec 6;9:2872. doi: 10.3389/fmicb.2018.02872 (PMC6291488; doi:10.3389/fmicb.2018.02872)

wgMLST (<All Characters>)

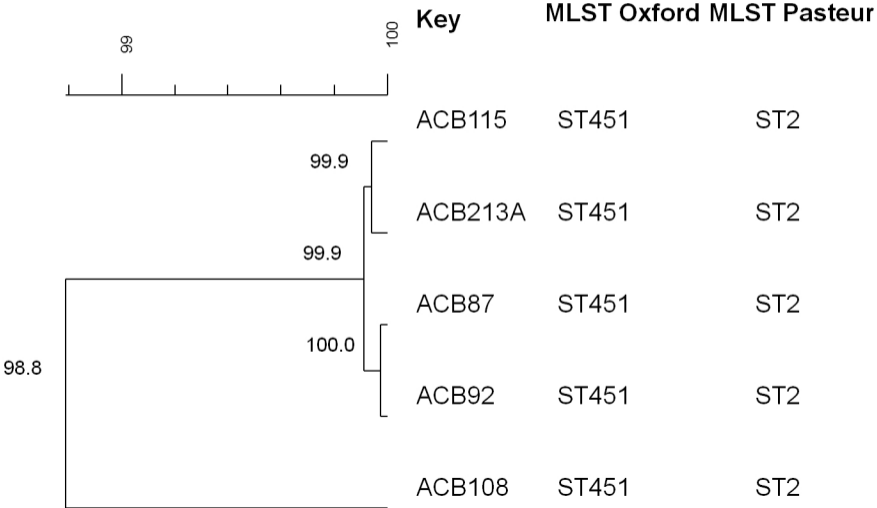

Supplement: Supplementary file 3 [file Image_1.PDF]

ABa87    Aba108    Aba213a  
Aba92    Aba115

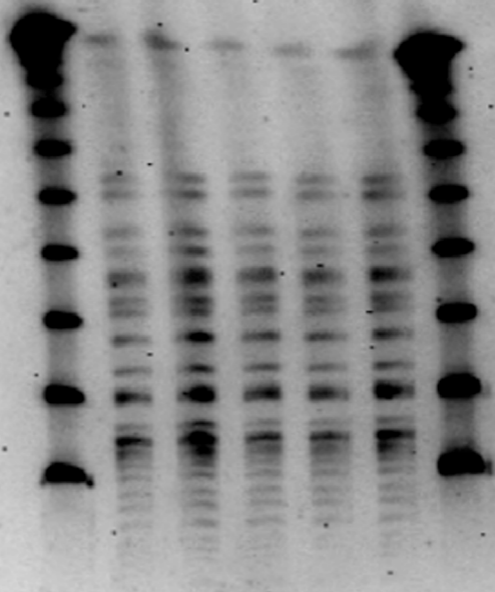

Supplement: Supplementary file 4 [file Image_2.PDF]
