# Supplementary material for: Hospitalized Pets as a Source of Carbapenem-Resistance
Source: Front Microbiol. 2018 Dec 6;9:2872. doi: 10.3389/fmicb.2018.02872 (PMC6291488; doi:10.3389/fmicb.2018.02872)
Supplement: Supplementary file 5 [file Table_1.docx]

**Table S1:** Characteristics of the isolates resistant to carbapenems and pertinent clinical data of the animals.

| **Isolate** | **Organism** | **Dog/Cat (source)** | **Sex**  **M/F** | **Age**  **(Years)** | **Veterinary Hospital** | **Sampling day** | **previous**  **antibiotic treatment/s** |
| --- | --- | --- | --- | --- | --- | --- | --- |
| **1A IMI** | ***Acinetobacter radioresistens*** | Dog | F | 7 | H1 | **02/10/2014** | **AMC/DO** |
| **87A IMI** | ***Acinetobacter baumannii*** | Cat | M | 1 | H2 | **11/10/2014** | **AMC** |
| **92A IMI** | ***Acinetobacter baumannii*** | Dog | F | 8 | H2 | **11/10/2014** | **AMC** |
| **108A IMI** | ***Acinetobacter baumannii*** | Cat | F | 4 | H2 | **26/10/2014** | **AMC** |
| **115A IMI** | ***Acinetobacter baumannii*** | Dog | M | 7 | H2 | **11/11/2014** | **AMC/EFX** |
| **213A IMI** | ***Acinetobacter baumannii*** | Cat* | F | 9 | H2 | **01/02/2015** | **AMC** |
| **3 A IMI** | ***Pseudomonas aeruginosa*** | Dog | M | 10 | H1 | **02/10/2014** | **SAM** |
| **110A IMI** | ***Pseudomonas aeruginosa*** | Dog | F | 10 | H2 | **04/11/2014** | **AMC** |
| **111A IMI** | ***Pseudomonas aeruginosa*** | Dog | F | 8 | H2 | **04/11/2014** | **PGS/AMC** |
| **117A IMI** | ***Pseudomonas aeruginosa*** | Dog | M | 6 | H2 | **15/11/2014** | **AMC/EFX/MTR/** |
| **121A IMI** | ***Pseudomonas aeruginosa*** | Dog | F | 15 | H2 | **17/11/2014** | **AMC/MTR** |
| **131A IMI** | ***Pseudomonas aeruginosa*** | Cat | F | 14 | H2 | **23/11/2014** | **AMC** |
| **207A IMI** | ***Stenotrophomonas maltophilia*** | Dog | F | 10 | - | **31/01/2015** | **ND** |
| **213B IMI** | ***Stenotrophomonas maltophilia*** | Cat* | F | 9 | H2 | **01/02/2015** | **AMC** |

SAM, ampicillin–sulbactam; AMC, Amoxicillin + Clavulanic Acid; DO, Doxycycline; EFX, Enrofloxacin; MTR, Metronidazole; PGS, Penicillin G + Dihydrostreptomycin; ND, Not done.
