# Supplementary material for: Hospitalized Pets as a Source of Carbapenem-Resistance
Source: Front Microbiol. 2018 Dec 6;9:2872. doi: 10.3389/fmicb.2018.02872 (PMC6291488; doi:10.3389/fmicb.2018.02872)
Supplement: Supplementary file 6 [file Table_2.docx]

**Table S2:** Resistance to antibiotics determined using disk diffusion following Clinical and Laboratory Standards Institute standard (CLSI 2014 M100-S24).

| **Strain** | **Organism** | SAM | CAZ | CTX | ATM | FEP | CIP | TET | GEN | AMK | SXT |
| --- | --- | --- | --- | --- | --- | --- | --- | --- | --- | --- | --- |
| **1** | ***Acinetobacter radioresistens*** | S | R | R | ND | R | R | R | S | S | R |
| **87** | ***Acinetobacter baumannii*** | R | R | R | ND | R | R | R | R | R | I |
| **92** | ***Acinetobacter baumannii*** | R | R | R | ND | R | R | R | R | R | R |
| **108** | ***Acinetobacter baumannii*** | R | R | R | ND | R | R | R | R | R | R |
| **115** | ***Acinetobacter baumannii*** | R | R | R | ND | R | R | R | R | R | I |
| **213A** | ***Acinetobacter baumannii*** | R | R | R | ND | R | R | R | R | R | I |
| **3** | ***Pseudomonas aeruginosa*** | ND | S | ND | S | S | S | R | S | S | R |
| **110** | ***Pseudomonas aeruginosa*** | ND | S | ND | S | S | S | R | S | S | R |
| **111** | ***Pseudomonas aeruginosa*** | ND | S | ND | S | S | S | R | S | S | R |
| **117** | ***Pseudomonas aeruginosa*** | ND | S | ND | S | S | S | R | S | S | R |
| **121** | ***Pseudomonas aeruginosa*** | ND | S | ND | S | S | S | R | S | S | R |
| **131** | ***Pseudomonas aeruginosa*** | ND | S | ND | S | S | R | R | R | S | R |
| **207** | ***Stenotrophomonas maltophilia*** | ND | ND | ND | ND | ND | ND | ND | ND | ND | R |
| **213B** | ***Stenotrophomonas maltophilia*** | ND | ND | ND | ND | ND | ND | ND | ND | ND | R |

Abbreviations: SAM, ampicillin–sulbactam; CAZ, ceftazidime ; CTX, cefotaxime; ATM, aztreonam; FEP, cefepime; CIP, ciprofloxacin; TET, tetracycline; GEN, gentamicin; AMK, amikacin; SXT, sulfamethoxazole–trimethoprim; R, resistant; I, intermediate; S,sensitive; ND, not determined in CLSI 2014 M100-S24.
