# Supplementary material for: Hospitalized Pets as a Source of Carbapenem-Resistance
Source: Front Microbiol. 2018 Dec 6;9:2872. doi: 10.3389/fmicb.2018.02872 (PMC6291488; doi:10.3389/fmicb.2018.02872)
Supplement: Supplementary file 7 [file Table_3.DOCX]

**Table S3:** Resistome of the *Acinetobacter baumannii* isolates analyzed with Resistance Gene Identifier v.4.0.2 (<https://card.mcmaster.ca>) on draft genomes

| **ARO Term** | **Detection criteria** | **AMR gene family** | **Drug class** | |
| --- | --- | --- | --- | --- |
| adeI | protein homolog model | RND AEP | macrolide , cephalosporin, lincosamide , diaminopyrimidine , tetracycline , rifamycin , phenicol , fluoroquinolone , carbapenem | |
| OXA-23 | protein homolog model | OXA beta-lactamase | cephalosporin, penam | |
| abeS | protein homolog model | SMR AEP | aminocoumarin , macrolide | |
| armA | protein homolog model | 16S rRNA methyltransferase | aminoglycoside | |
| APH(3')-Ia | protein homolog model | APH(3') | aminoglycoside | |
| OXA-66 | protein homolog model | OXA beta-lactamase | cephalosporin, penam | |
| adeK | protein homolog model | RND AEP | macrolide , cephalosporin, lincosamide , diaminopyrimidine , tetracycline , rifamycin , phenicol , fluoroquinolone , carbapenem | |
| TEM-1 | protein homolog model | TEM beta-lactamase | penem, monobactam, cephalosporin, penam | |
| tetR | protein overexpression model | MFS AEP | glycylcycline, tetracycline | |
| adeS | protein homolog model | RND AEP | glycylcycline, tetracycline | |
| adeB | protein homolog model | RND AEP | glycylcycline, tetracycline | |
| adeN | protein homolog model | RND AEP | macrolide , cephalosporin, lincosamide , diaminopyrimidine , tetracycline , rifamycin , phenicol , fluoroquinolone , carbapenem | |
| adeF | protein homolog model | RND AEP | fluoroquinolone , tetracycline | |
| adeN | protein homolog model | RND AEP | macrolide , cephalosporin, lincosamide , diaminopyrimidine , tetracycline , rifamycin , phenicol , fluoroquinolone , carbapenem | |
| APH(3'')-Ib | protein homolog model | APH(3'') | aminoglycoside | |
| adeL | protein homolog model | RND AEP | fluoroquinolone , tetracycline | |
| ADC | protein homolog model | ADC beta-lactamase | cephalosporin | |
| adeJ | protein homolog model | RND AEP | macrolide , cephalosporin, lincosamide , diaminopyrimidine , tetracycline , rifamycin , phenicol , fluoroquinolone , carbapenem | |
| abeM | protein homolog model | MATET | triclosan, acridine dye, fluoroquinolone | |
| adeH | protein homolog model | RND AEP | fluoroquinolone , tetracycline | |
| ANT(3'')-IIa | protein homolog model | ANT(3'') | aminoglycoside | |
| tet(A) | protein homolog model | MFS AEP | glycylcycline, tetracycline | |
| adeR | protein homolog model | RND AEP | glycylcycline, tetracycline | |
| adeG | protein homolog model | RND AEP | fluoroquinolone , tetracycline | |
| APH(6)-Id | protein homolog model | APH(6) | aminoglycoside | |
| adeA | protein homolog model | RND AEP | glycylcycline, tetracycline |  |

ARO: Resistance Ontology; AMR, antimicrobial resistance; RND, resistance-nodulation-cell division; AEP, efflux pump; MFS, major facilitator superfamily ;MATET, multidrug and toxic compound extrusion transporter; SMR, small multidrug resistance.
